# Understanding the Hierarchical Relationships in Female Sex Workers’ Social Networks Based on Knowledge, Attitude, and Practice

**DOI:** 10.3390/ijerph16203841

**Published:** 2019-10-11

**Authors:** Xianlei Dong, Mengge Sun, Jingguo Wang, Zhihan Yang, Beibei Hu

**Affiliations:** 1 School of Business, Shandong Normal University, Jinan 250358, China; sddongxianlei@163.com; 2 National Science Library, Chinese Academy of Science, Beijing 100190, China; menggesun1997@163.com; 3 Department of Library Information and Archives Management, UCAS, Beijing100039, China; 4 Information Systems and Operation Management, College of Business, University of Texas at Arlington, Arlington, TX 76019, USA; JWANG@uta.edu; 5 Statistics with Data Science, School of Mathematics, The University of Edinburgh, Edinburgh EH8 9YL, UK; zhihanyang1997@163.com

**Keywords:** hierarchical social network, knowledge, attitude, and practice (KAP), HIV/AIDS, individual character

## Abstract

Female sex workers (FSWs) represent a high-risk population for HIV infection and transmission. In general, their fellow FSWs (peers) also play a role in their level of susceptibility to HIV/AIDS. This paper draws from interview data of 93 FSWs to construct a multi-layer FSW social network model based on their knowledge, attitude, and practice (KAP). Statistical analyses of the correlation among the three dimensions of KAP as well as their social interactions indicated that FSWs had basic knowledge of HIV/AIDS prevention but demonstrated little enthusiasm in acquiring relevant information. Their knowledge, attitude, and practice were highly positively correlated. Their attitude was more likely to be negatively influenced by their companions, while their practice was more likely to be positively affected. Besides, FSWs exhibited high homophily in KAP with their neighbors. Thus, during HIV/AIDS interventions, FSWs should receive individualized education based on their specific KAP. Considering the high level of homophily among FSWs, their propensity to be positive or negative in their KAP are significantly influenced by their companions. Making full use of peer education and social interaction-based interventions may help prevent and control the spread of HIV/AIDS.

## 1. Introduction

In China, heterosexual behavior is one of the main modes of human immunodeficiency virus (HIV) transmission [1]. In fact, the proportion of people who become infected due to the fact of heterosexual behavior has increased over the last decade. In 2007, it was the cause of only 36.1% of cases [2], while in 2017 (as of 31 October), that figure reached 68.50% [3]. This large increase may be somewhat attributed to the behaviors of female sex workers (FSWs)—women who make a living by providing paid sexual services. They are a high-risk population for HIV infection and transmission because they have multiple sexual partners and engage in risky sexual behaviors (such as low rates of condom use during commercial sex) and, therefore, represent a key link in the spread of HIV from high-risk groups to the general population [3,4].

According to the literature, active sexual behavior, limited education, and sensitive interpersonal relationships characterize most FSWs [5,6]. Indeed, a majority lack knowledge about HIV/AIDS prevention, have a negative attitude toward it [7], often do not use condoms, and engage in high-risk sexual behaviors [8,9,10,11]. Therefore, FSWs, especially those working in low-end places, such as shampooing rooms, beauty parlors or sauna rooms, would benefit from HIV prevention outreach services [12,13,14,15].

Referring to previous studies, the knowledge–attitude–practice (KAP) model can provide insight into FSW health behaviors [16]. According to this framework, an individual’s knowledge about a disease positively affects their attitude toward prevention and eventually changes their practices. Additionally, KAP is one of the cornerstones in the fight against HIV/AIDS—gaining knowledge about the disease promotes positive attitudes toward prevention and safe sex practices [16,17]. For example, in the field of public health and preventive medicine, this model is often used to measure HIV protection status among FSWs and to evaluate the intervention effects [18]. Initiatives based on the KAP model, are proposed to increase knowledge about HIV/AIDS, change attitudes toward prevention, and reduce risky behavior [16,17]. Therefore, assessing KAP among FSWs is important in HIV/AIDS prevention and helps evaluate the success of prevention strategies [19,20].

Based on the KAP model, many intervention programs have focused on disseminating information about HIV/AIDS to dispel misconceptions and promote safe practices. In general, education and intervention programs for FSWs mainly comprise distributing educational materials, providing HIV/AIDS-related information, promoting condom use, and HIV screening and treatment [21,22,23]. These projects based on the KAP model contribute a lot to the HIV/STI intervention. For example, a related program in China found that the awareness rate of principal knowledge about HIV/AIDS by FSWs increased by 4.88% on average after two years of KAP intervention [12]. It has been proved that peer education is a very efficient intervention method. In the same program, when the FSWs were offered peer education 1~3 times, their awareness rate became 2.426 times higher than before [12].

Another important factor to consider within the framework of the KAP model is social relations, which play a role in an individual’s KAP level [24]. A social interaction network refers to social connections, such as friends and acquaintances, and may help explain the similarities and differences among them in terms of HIV/AIDS prevention [25,26,27,28,29]. The topological structure of a social network and key FSWs could have a strong influence on the level of engagement in safe practices that prevent HIV transmission [30]. Based on the structural characteristics of FSW social networks, the efficiency of intervention programs, such as educational campaigns, may be improved [31,32,33,34,35].

In the study of raising the awareness of HIV prevention among FSWs, some previous studies isolated the FSWs group into individuals and paid great attention to individual attribute characteristics [19,20,21,22]. In other words, previous studies based on individual-level analyses could not systematically show the inter-correlations among friends and acquaintances [36]. Meanwhile, studies on FSW social networks explored only the structural characteristics of FSWs but not the inter-correlations of individual characteristics [37]. In this paper, we established a multi-layer FSW social network based on the social connections among FSWs and their knowledge, attitude, and practice related to HIV/AIDS. Then, we proposed a probability model to quantify peers’ impacts in the multi-layer network. Doing so provides a novel way to study the relationships between individual characteristics and social influence among FSWs.

We conducted face-to-face interviews and asked questions to not only assess knowledge, attitudes, and practices related to HIV/AIDS (i.e., AIDS KAP questionnaire) but to also find out about their relationships to other FSWs. This is in line with other information collection and HIV/AIDS studies wherein AIDS KAP questionnaires are widely used [24,38]. We referenced the AIDS Indicator Survey model [39,40] and previous AIDS KAP questionnaires [41] to design ours to suit our target samples. This study aimed to understand the influence other FSWs (their companions) have on the individual in terms of knowledge, attitude, and practice.

This paper is organized as follows: Section 2 describes data collection and methodology, including the multi-layer FSW network model, Section 3 discusses the hierarchical relationships in the multiple social networks of FSWs, from the perspective of individual effect and social influence, and Section 4 concludes the study.

## 2. Materials and Methods

### 2.1. Data

We collected data through one-on-one interviews with FSWs at different locations in a city in Yunnan province, China, between November 2014 and April 2015. First, we randomly chose a number of workplaces to visit and asked the shop owner to agree to interviews. The venues included bars, hair salons, karaoke halls, massage parlors, nightclubs, and hotels. A stratified random sampling method was used to reach these workplaces based on their distribution of workplace counts in that city to avoid a sample bias [41,42]. Then, we interviewed all the FSWs who were there but not in service at that time, one by one. We spent approximately three months collecting the FSWs’ responses to the questions (see Table A1) related to HIV/AIDS knowledge, attitude, and practice (KAP). We referred to the AIDS Indicator Survey and previous AIDS KAP questionnaires and adapted them for FSWs in regard to Chinese cultural and ethnic customs. We also interviewed the shop owners (most were also FSWs) whom the other FSWs worked for. Then, it took us an additional three months to acquire the acquaintance relationships among these FSWs (i.e., whether they have social interactions with each other). In total, we gathered 93 responses from 10 workplaces.

As a limitation, we realized that the sample size was relatively small, and it may not be representative. Thus, our research may be illustrative in nature. However, the small sample seems quite common in similar topics due to the difficulty in data collection through surveys and interviews. For example, Battiston (2014) [43] proposed a similar theoretical method, namely, conditional probability combined with a complex network structure to study the existence of correlations across the layers of a multiplex using 78 Indonesian terrorists. Another study (Tuchker et al., 2011) [44] used 62 FSWs to examine how social relationships affect their behavior toward preventing HIV.

### 2.2. Methodology

#### 2.2.1. Division of Individual Characteristics

##### Interview Questions

In the AIDS KAP questionnaire, questions on knowledge include understanding of HIV/AIDS, transmission, and risk factors; questions on attitudes include voluntarily taking part in HIV/AIDS prevention training or taking the initiative to understand HIV/AIDS prevention; and finally, questions on practices include safety measures and high-risk sexual behaviors. Table 1 shows a small sample of the responses from the 93 FSWs interviewed (refer to Table A1 for a complete list).

Most FSWs knew some information about HIV/AIDS. They had a basic understanding of HIV transmission routes, prevention practices, and the associated risk. However, over half still had some misconceptions. For example, 63 respondents mistakenly believed mosquito bites can transmit HIV. Although a few had proactively sought out information on HIV/AIDS (e.g., through radio, television, traditional media, etc.), most were quite passive in HIV/AIDS prevention. Most had only sought out a few voluntary HIV tests and did not participate in public service activities, such as free HIV/AIDS counseling, free condoms or prevention skills training (only 1 of the 93 FSWs).

##### The Division of Node Attribute

The primary goal of the HIV/AIDS intervention program is to convert “negative” FSWs into “positive” ones, or in other words, help FSWs have more knowledge, have fewer misconceptions, and engage in safer sexual practices in terms of HIV/AIDS transmission. The more questions answered correctly, the more positive she is, and the more questions answered incorrectly, the more negative she is. According to our previous study [45], we used Z-scores to identify the relative positivity or negativity of FSWs based on their respective responses to KAP questions. This standardization method measures the distance between an individual and the mean; thus, a relatively large Z-score often indicates an individual has answered more questions correctly, i.e., the individual is more positive. Please note that we use the terms “negative” and “positive” in a relative sense as worse or better, but not in an absolute term.

Counting FSWs’ responses to all the interview questions in one category (or attribute layer α, here  α=K,A,P; where K,A and P denote knowledge, attitude and practice respectively), let $T_{i}^{\left[\alpha \right]}$ denote the number of positive responses from $FSW_{i}$ (i= 1,2,…,93); $u^{\left[\alpha \right]}$ the average number of positive responses of all FSWs in attribute layer α; and $\sigma^{\left[\alpha \right]}$. the standard deviation. We used the following Equation (1) to calculate the Z-scores of all FSWs in layer α:

(1) $$Z_{i}^{\left[\alpha \right]}=\frac{T_{i}^{\left[\alpha \right]}-u^{\left[\alpha \right]}}{\sigma^{\left[\alpha \right]}}$$

when $Z_{i}^{\left[\alpha \right]}\geq 0$, we consider the FSW to be positive; otherwise, she is considered negative. It is worth pointing out that, although we subjectively distinguished FSWs into positive ones and negative ones according to their average performance, we actually mean that positive FSWs are more active than negative ones, which is not an absolute judgment.

#### 2.2.2. Construction of Multi-Layer FSW Social Network Model

##### FSW Social Network

**Figure 1:**
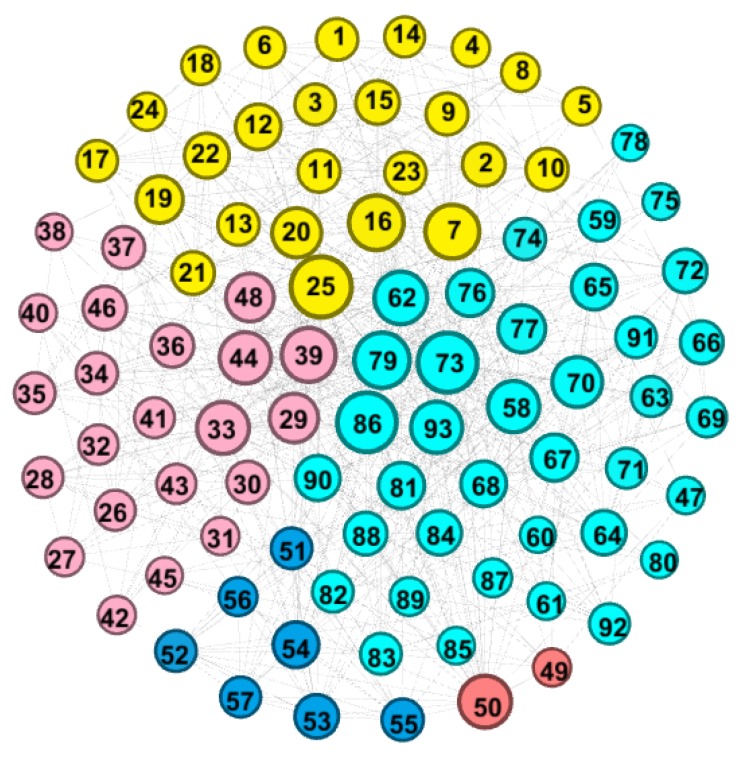
Topology of the female sex worker (FSW) social network.

We built the social interaction network of FSWs based on acquaintance relationships. The network designates FSWs as nodes and the social relations among FSWs (acquaintance or not) as edges (relationship). The FSW social network can be represented by a graph *G*(*N*, *A*), which consists of nodes set *N* and edges set *A*. Among them, each FSW is uniquely represented by a node ID number, and the edge set could be translated into an adjacent matrix $K=\left\{k_{ij}\right\}\;$, in which $k_{ij}$ represents the social relationship between node *i* and node *j*. If *i* and *j* are acquaintances, then they are directly connected and $k_{ij}=1\;$; otherwise $\;k_{ij}=0\;$. We visualized the network in Figure 1 below.

As shown in Figure 1, we divided the FSW nodes into five sections, each one a different color to represent a different type of workplace. Nodes 1 to 25 work in a bar or karaoke hall, 26 to 48 a salon, 49 and 50 a sauna or massage parlor, 51 to 57 nightclubs or hotels, and 58 to 93 other workplaces. The node size reflects the amount of social connections the FSW has in the network; thus, larger ones represent FSWs who know a larger number of their peers in this study. On another level, the size could also be a reflection of one’s social skills and importance to the entire network.

##### Multi-Layer FSW Social Network

**Figure 2:**
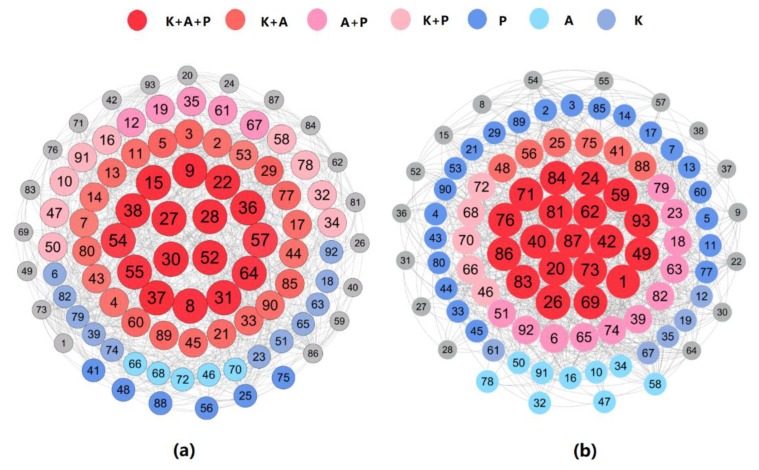
Multi-layer FSW social network. K, knowledge, A, attitudes, P, practices.

Next, we built a weighted multi-layer FSW social network based on (1) the social relations among FSWs and (2) their positive and negative Z-scores at different layers. We calculated the weight of a node in the multi-layer network as follows: First, when node i was part of a positive group in the  α layer (i.e., $Z_{i}^{\left[\alpha \right]}\geq 0$), then$\;a_{i}^{\left[\alpha \right]}=1$; otherwise $a_{i}^{\left[\alpha \right]}=0$. Then, we defined the positive weight of node  i as $W_{i}=\sum_{\alpha }\;a_{i}^{\left[\alpha \right]}$. Accordingly, ${\bar{W}}_{i}\;=\;3\;-\;W_{i}$ represents the negative weight of node i. Based on the positive and negative weight of node i, Figure 2 illustrates the multi-layer FSW social network.

Figure 2a,b are two different expressions of the multi-layer FSW social network. More specifically, Figure 2a is a FSW social network based on positive weight ($W_{i}$), representing the positive tendency of the nodes, while Figure 2b is based on negative weight (${\bar{W}}_{i}$), representing the negative tendency of the nodes. For example, in Figure 2a, the red-colored nodes are positive in all three layers, while in Figure 2b, the light blue ones are negative in the practice layer and positive in the knowledge and attitude layers. As for size, in Figure 2, it reflects the scale of the node’s absolute value. That is, a larger node indicates this FSW is more positive (in Figure 2a) or negative (in Figure 2b).

As we can see from Table 2 and Figure 2, of the 93 FSWs, 16 and 19 have three positive or negative Z-scores, respectively, while 58 have both positive and negative Z-scores in different layers. In addition, 22 FSWs have positive Z-scores in a single layer and negative in the other two layers. In total, most FSWs are positive in at least one layer.

However, the shop owners, in particular, have special features in the multi-layer FSW social network. In fact, 33.3%, 11.1%, and 55.6% are negative in three layers, two layers, and a single layer, respectively. In other words, none of the shop keepers are positive in all the three layers, while 19.5% of the other FSWs are, in contrast. Compared to the latter, they tend to be even more negative in terms of HIV/AIDS prevention. Considering their wider range of social connections and greater degree of influence, they may have a more significant negative impact on their acquaintances than the non-shop keeper FSWs do. Thus, it is especially important to reach out and educate shop owners.

##### Positive and Negative FSW Social Sub-Networks

**Figure 3:**
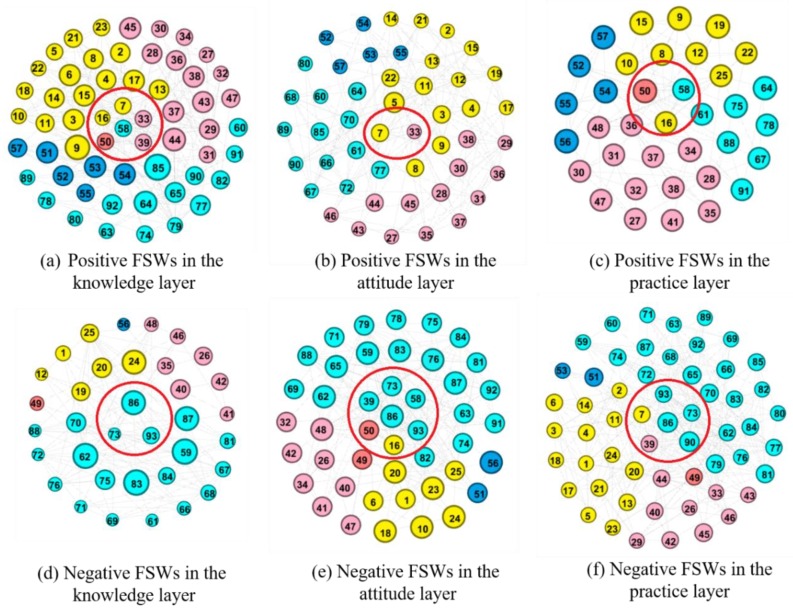
Positive and negative FSW social sub-networks.

To further analyze the interaction of the FSWs, we extracted the weighted positive and negative sub-network in the knowledge, attitude, and practice layers, respectively, from the multi-layer FSW social network. Let $T_{i}^{\left[\alpha \right]}$ denote the number of positive responses of node i in  α layer, and $F_{i}^{\left[\alpha \right]}$ the number of negative responses. Second, let $R_{i}^{\left[\alpha \right]}$, as shown in Equation (2), denote the weight of node  i in positive sub-network of α layer. As $R_{i}^{\left[\alpha \right]}$ increases, the FSW is more positive. Let ${\bar{R}}_{i}^{\left[\alpha \right]}$, as shown in Equation (3), denote the weight of node  i in the negative sub-network of the α layer. As ${\bar{R}}_{i}^{\left[\alpha \right]}$ increases, the FSW is more negative. Figure 3 lists the extracted positive and negative sub-networks. Figure 3a–c represent the positive sub-networks of the knowledge, attitude, and practice layers, respectively, while Figure 3d–f represent the corresponding negative sub-networks. Different workplaces and the positive (negative) level of nodes are respectively distinguished by color and size. The nodes in the red circles are shop keepers.

(2) $$R_{i}^{\left[\alpha \right]}=T_{i}^{\left[\alpha \right]}-F_{i}^{\left[\alpha \right]}$$

(3) $${\bar{R}}_{i}^{\left[\alpha \right]}\;=F_{i}^{\left[\alpha \right]}-\;T_{i}^{\left[\alpha \right]}$$

Compared with the FSWs working in a salon, bar or karaoke hall, those working in high-end venues, such as nightclubs and hotels, tend to be more positive, as shown in Figure 3 and Table 3. For instance, among FSWs working in nightclubs and hotels, 85.7, 71.4, and 71.4 percent are positive in the knowledge, attitude, and practice layers, respectively, while among FSWs working in a bar/salon, they are 76/72.7, 64/63.6, and 36/59.1 percent, respectively. Among the few FSWs working at other venues, 44.4, 36.1, and 22.2 percent are positive in the knowledge, attitude, and practice layers, respectively. In addition, only 66.7, 22.2, and 33.3 percent of shop owners are positive in the knowledge, attitude, and practice layers, respectively. Thus, they are more positive in the knowledge layer but more negative in the attitude and practice layers.

## 3. Results

### 3.1. Measuring the Association between Responses to Knowledge, Attitude, and Practice

First, we used a probabilistic method (i.e., conditional probability) to measure the association of an individual’s responses to KAP. Let $a_{i}^{\left[\alpha \right]}=1$ when node i is part of a positive group in  α layer; otherwise $a_{i}^{\left[\alpha \right]}=0$. Let $\alpha^{\prime }$ denote the complementary set of α. For example, let $\;\alpha^{\prime }=A,P$ when α = K. On the condition that node i is part of a positive group in  α layer, the probability that node  i is also part of a positive group in $\alpha^{\prime }$ layer is as shown in Equation (4):

(4) $$p\left(a_{i}^{\left[\alpha^{\prime }\right]}/a_{i}^{\left[\alpha \right]}\right)=\frac{\sum_{i}a_{i}^{\left[\alpha \right]}a_{i}^{\left[\alpha^{\prime }\right]}}{\sum_{i}a_{i}^{\left[\alpha \right]}}$$

**Figure 4:**
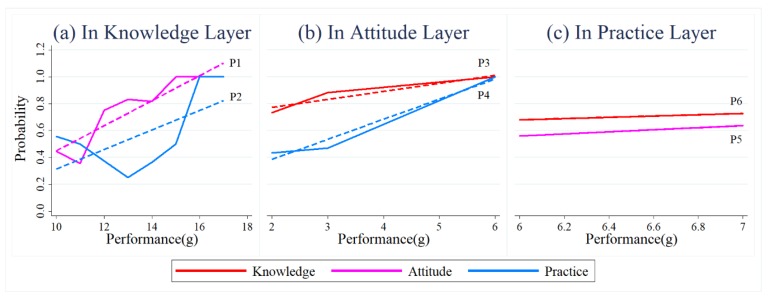

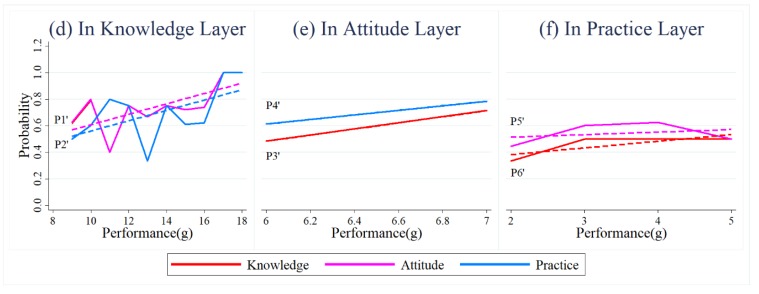
The relationships of individuals’ performances at different layers. The solid and dashed lines, respectively, represent true and linear fitted values. The *x*-axes refer to the performance of the FSWs in $O_{g}^{\left[\alpha \right]}$, who have the same positive (**a**–**c**) or negative (**d**–**f**) responses on the questionnaire in the  α layer. The *y*-axes refer to the proportion of these FSWs in subset $O_{g}^{\left[\alpha \right]}$ also being positive (**a**–**c**) or negative (**d**–**f**) in the other two layers. Please refer to Table A2 for the fitted equations and parameters of the models as well as the results of significant testing. We only provided the conclusions when the fitted lines passed the related significant testing.

Let $O^{\left[\alpha \right]}$ denote the set of positive FSWs in the  α layer, and $\left\{O_{g}^{\left[\alpha \right]}\left(g=1,2,\ldots ,n^{\left[\alpha \right]}\right)\right\}$ is a partition of $O^{\left[\alpha \right]}$, wherein $n^{\left[\alpha \right]}$ denotes the total number of questions about α(α=K,A,P) in the questionnaire. $O_{g}^{\left[\alpha \right]}$ denotes the subset of FSWs who have the same performance g, which refers to the number of positive responses about  α in the questionnaire. As g increases, the subset $O_{g}^{\left[\alpha \right]}$ becomes more positive. Then, we used Equation (5) to calculate the probabilities of positive node i in subset $O_{g}^{\left[\alpha \right]}$ also being positive in layer $\alpha^{\prime }$, as shown in Figure 4a–c. Figure 4a–c represent the correlations of positive individuals’ performance in KAP when  α is knowledge, attitude and practice, respectively. Similarly, we also used the same equation to calculate the probabilities, of negative node  i also being negative in layer $\alpha^{\prime }$ as shown in Figure 4d–f. Figure 4d–f represent the correlations of negative individuals’ responses to KAP when  α is knowledge, attitude, and practice, respectively.

(5) $$P\left(a_{i}^{\left[\alpha^{\prime }\right]}/a_{i}^{\left[\alpha \right]}\right)=\frac{\sum_{j\in O_{g}^{\left[\alpha \right]}}a_{j}^{\left[\alpha \right]}a_{j}^{\left[\alpha^{\prime }\right]}}{\sum_{j\in O_{g}^{\left[\alpha \right]}}a_{j}^{\left[\alpha \right]}}$$

In general, the results indicated a strong positive correlation among responses in the knowledge, attitude, and practice layers. For a node, the more positive an FSW was in  α layer, the higher the probability she was positive in the other two layers. For example, the more positive a node was in the attitude layer, the higher probability of being positive in the knowledge and practice layers (as shown in Figure 4b). With the increase of the probability of nodes being positive in the knowledge layer, the probability of being positive decreased before rising in the other two layers (Figure 4a). In particular, there were several fluctuations when g = 11 and g = 13 in the knowledge layer. The key reason was that the subsets of$\;O_{11}^{\left[K\right]}$ and $O_{13}^{\left[K\right]}$ mainly included FSWs who worked in a bar, karaoke hall, and other venues. The FSWs in those workplaces were characterized by positive knowledge, but negative attitude and practices. Meanwhile, when g = 5, the inclusion of shop owners (half of the FSWs in this group) resulted in a decreasing tendency of being negative in the knowledge layer (as shown in Figure 4f); they generally exhibited positive knowledge but negative practice.

In addition, an increase in a node’s negativity in the attitude layer led to an increase in negativity in the practice layer. However, it is difficult to determine whether the node tended to be negative or not in the attitude layer alongside an increase in negativity in the practice layer. This indicates that attitude can affect practice, but the reverse may not be true. Moreover, other factors besides attitude, such as workplace requirements, may influence behavior.

### 3.2. Measuring Neighbor Impact on an Individual in a Single Layer

A FSW’s companions—her neighbor nodes in the social network—may influence whether she is positive in a layer. To determine this, we measured neighbors’ impact on an individual in a certain layer. We started from the basic assumption that communication among people can affect each individual’s knowledge, attitude, and practices. Then, neighbors’ impact on an individual in a certain layer was positively correlated to, on the one hand, the number of her neighbors (i.e., node degree) and, on the other hand, the positive or negative responses of her neighbors (i.e., the neighbors’ positive or negative weights) [28]. For example, an FSW would be positively influenced when she has a lot number of friends who have lots of HIV/AIDS knowledge and are very positive on attitude and behavior. Therefore, we looked into a FSW’s social environment in a layer based on her neighbors’ responses in the same layer, using the sum of weights of all her neighbors as defined below.

For node i in α layer, let $p_{i}^{\left[\alpha \right]}$ denote an impact index from positive neighbors, as shown in Equation (6). For neighbors *j* of node i, $R_{j}^{\left[\alpha \right]}$ denotes the weight of positive node *j* in the positive sub-network of the  α layer. A relatively large weight indicates that node *j* is more positive and has a more significant impact.

(6) $$p_{i}^{\left[\alpha \right]}=\underset{j}{\sum }R_{j}^{\left[\alpha \right]}=\underset{j}{\sum }\left(T_{j}^{\left[\alpha \right]}\;-\;F_{j}^{\left[\alpha \right]}\right)$$

Similarly, for node i in the  α layer, let $n_{i}^{\left[\alpha \right]}$ denote the impact index from negative neighbors in the same layer, as shown in Equation (7). For the neighbors *j* of node i, ${\bar{R}}_{j}^{\left[\alpha \right]}$ denotes the weight of negative node *j* in the negative sub-network of  α layer. A relatively large weight indicates the node *j* is more negative and has a more significant impact.

(7) $$n_{i}^{\left[\alpha \right]}=\underset{j}{\sum }{\bar{R}}_{j}^{\left[\alpha \right]}=\underset{j}{\sum }\left(\;F_{j}^{\left[\alpha \right]}\;-\;T_{j}^{\left[\alpha \right]}\right)$$

Let$\;s_{i}^{\left[\alpha \right]}$ denote the impact index of node *i* from all of her positive and negative neighbors, as shown in Equation (8).

(8) $$\;s_{i}^{\left[\alpha \right]}=p_{i}^{\left[\alpha \right]}\;-\;n_{i}^{\left[\alpha \right]}$$

$\;s_{i}^{\left[\alpha \right]}\;>\;0\;$ implies positive neighbors have stronger effects on node i. In other words, neighbors positively affect node i. $\;s_{i}^{\left[\alpha \right]}\;<\;0\;$ indicates negative neighbors have a stronger effect on node i, i.e., neighbors negatively affect node i. In fact, $\;s_{i}^{\left[\alpha \right]}$ measures the potential influence from all neighbors in the  α layer on node i.

**Figure 5:**
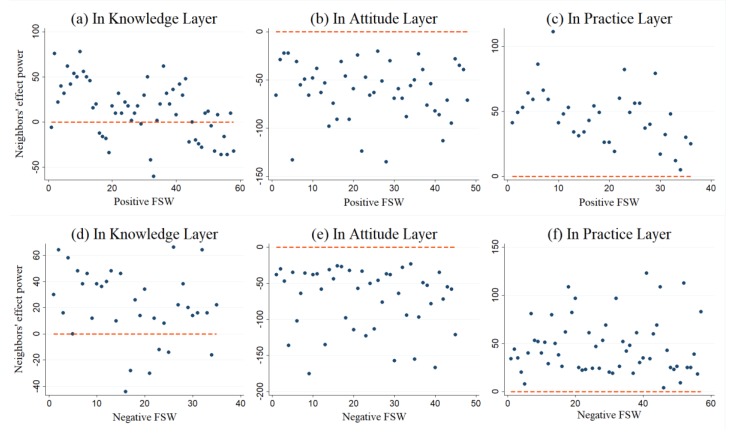
Positive or negative impact power from FSWs’ neighbors. The *x*-axes refer to the positive FSWs (a-c) or negative FSWs (d-f). The *y*-axes refer to their neighbors’ effect power $\;s_{i}^{\left[\alpha \right]}$ of FSW i. The red dashed lines in the figure are the auxiliary reference line with $\;s_{i}^{\left[\alpha \right]}\;=\;0$.

Figure 5 describes the positive and negative impact of neighbors on the knowledge, attitude, and practice layers, respectively. Figure 5a–c represent neighbors’ impact indices on positive nodes, while Figure 5d–f represent neighbors’ impact indices on negative nodes.

**Figure 6:**
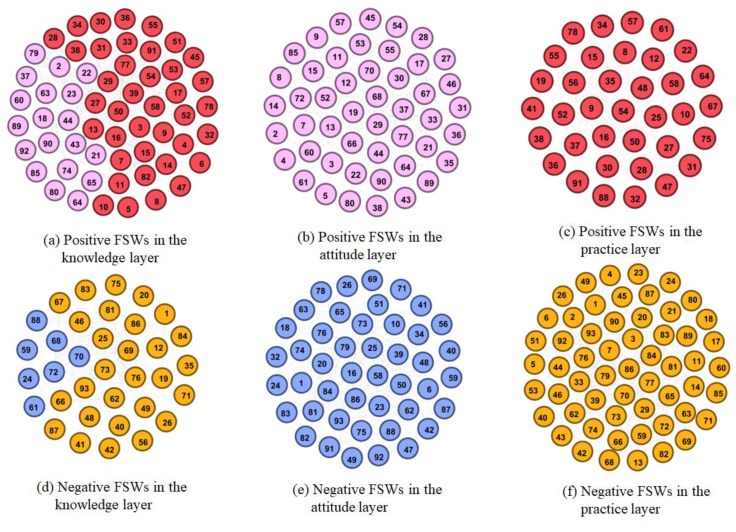
Positive or negative effects of neighbors. (**a**–**c**) Represent the positive sub-network in the KAP layers. Red nodes are positively influenced by neighbors in the same layer, while purple nodes are negatively influenced. (**d**–**f**) Represents the negative sub-network, with orange indicating the node is positively influenced by neighbors and blue the opposite.

As shown in Figure 5 and Figure 6, neighbors are more likely to negatively influence attitude than practice, while knowledge and practice are more likely to be positively influenced than attitude. Especially in the attitude layer, FSWs are not as positively affected by their neighbors (i.e.,$\;\;s_{i}^{\left[\alpha \right]}\;<\;0$) (as shown in Figure 5b,e and Figure 6b,e). Meanwhile, as shown in Figure 5c,f and Figure 6c,f, FSWs in the practice layer are not as negatively affected by their neighbors (i.e.,$\;\;s_{i}^{\left[\alpha \right]}\;>\;0$). In sum, FSWs are more likely to be positively influenced in the knowledge and practice layers and negatively influenced in the attitude layer.

### 3.3. Measuring Neighbors’ Influence on an Individual in the Multi-Layer Network

Neighbors influenced FSWs in other layers as well as in the same layer. Therefore, we used a probabilistic method to measure the overall effect of neighbors on an individual in a certain layer, namely, how a FSW’s responses were correlated with that of her neighbors—for instance, for a positive FSW in the knowledge layer, how her attitude and practice were correlated with her neighbors’ responses.

#### 3.3.1. Positive and Negative Neighbors

This is how we defined positive and negative nodes on the multi-layer network. First, let $T_{i}=\frac{T_{i}^{\left[K\right]}}{n^{\left[K\right]}}+\frac{T_{i}^{\left[A\right]}}{n^{\left[A\right]}}+\frac{T_{i}^{\left[P\right]}}{n^{\left[P\right]}}$ denote the overall positive level of node i. $T_{i}^{\left[\alpha \right]}\left(\alpha =K,A,P\right)$ represents the positive level of node i in α layer, while $n^{\left[\alpha \right]}$ denotes the total number of questions about  α in the questionnaire. Then, by calculating the Z-score of $T_{i}$, we identified the FSWs as positive or negative nodes. When $Z_{i}\geq 0$, we consider an FSW to be positive and otherwise negative.

#### 3.3.2. Neighbors’ Influence on Positive FSWs

For a positive FSW in the α layer, we studied her responses in the other layers to consider her neighbors’ influence. In other words, for a positive FSW in a certain layer, we determined how her responses (i.e., positive/negative probability in a layer) were correlated with her neighbors’ overall responses. In the  α layer, let $q_{i}^{\left[\alpha \right]}$ denote the negative neighbor ratio of positive node i, as shown in Equation (9).

(9) $$q_{i}^{\left[\alpha \right]}=\frac{N_{i}^{negative}}{N_{i}^{positive}+N_{i}^{negative}}$$

Then 1−$q_{i}^{\left[\alpha \right]}$ is the positive neighbor ratio of positive node i. In addition, $N_{i}^{negative}$ and $N_{i}^{positive}$ denote the number of negative and positive neighbors of node i, respectively. Thus, all the positive nodes with the same $q_{i}^{\left[\alpha \right]}$ value form a subset $O_{q}^{\left[\alpha \right]}$. Then, let $q_{O_{q}^{\left[\alpha \right]}}^{\left[\alpha^{\prime }\right]}$ denote the ratio of being negative in $\alpha^{‘}$ layers for the nodes in $O_{q}^{\left[\alpha \right]}$, as shown in Equation (10), wherein $N_{O_{q}^{\left[\alpha \right]}}$ denotes the elements in subset $O_{q}^{\left[\alpha \right]}$, and $N_{O_{q}^{\left[\alpha \right]}}^{negative},\;N_{O_{q}^{\left[\alpha \right]}}^{positive}$ denote the negative and positive numbers in $\alpha^{‘}$ layers for the nodes in $O_{q}^{\left[\alpha \right]}$.

(10) $$q_{O_{q}^{\left[\alpha \right]}}^{\left[\alpha^{\prime }\right]}=\frac{N_{O_{q}^{\left[\alpha \right]}}^{negative}}{N_{O_{q}^{\left[\alpha \right]}}}$$

**Figure 7:**
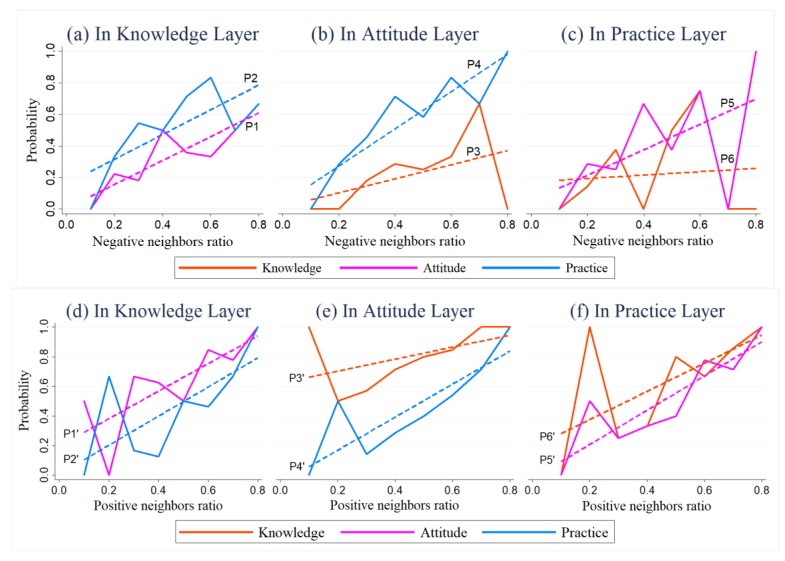
Negative (**a**–**c**) and positive (**d**–**f**) neighbors’ influence on positive FSWs. The solid and dashed lines represent the true values and the linear fitted values of possibility of $q_{O_{q}^{\left[\alpha \right]}}^{\left[\alpha^{\prime }\right]}$ and 1 − $q_{O_{q}^{\left[\alpha \right]}}^{\left[\alpha^{\prime }\right]}$, respectively. Please refer to Table A3 for the fitted equations and parameters of the models as well as the results of significant testing. We only provided the conclusions when the fitted lines passed the related significant testing.

Similarly, the positive ratio in the $\alpha^{‘}$ layers for the nodes in $O_{q}^{\left[\alpha \right]}$ is 1 − $q_{O_{q}^{\left[\alpha \right]}}^{\left[\alpha^{\prime }\right]}$. Figure 7a–c describe the correlations between $q_{i}^{\left[\alpha \right]}$ and $q_{O_{q}^{\left[\alpha \right]}}^{\left[\alpha^{\prime }\right]}$, while Figure 7d–f describe the correlations between 1 − $q_{i}^{\left[\alpha \right]}$ and 1 − $q_{O_{q}^{\left[\alpha \right]}}^{\left[\alpha^{\prime }\right]}$. The *x*-axis represents $q_{i}^{\left[\alpha \right]}$ (Figure 7a-c) or 1 − $q_{i}^{\left[\alpha \right]}$ (Figure 7d–f) (i.e., the negative or positive neighbor ratio of positive node i), while the *y*-axis represents $q_{O_{q}^{\left[\alpha \right]}}^{\left[\alpha^{\prime }\right]}$ (Figure 7a–c) or 1 $-\;q_{O_{q}^{\left[\alpha \right]}}^{\left[\alpha^{\prime }\right]}$ (Figure 7d–f) (i.e., the ratio of being negative or positive in $\alpha^{‘}$ layers for the nodes in $O_{q}^{\left[\alpha \right]}$). Figure 7a–c represent the correlations between $q_{i}^{\left[\alpha \right]}$ and $q_{O_{q}^{\left[\alpha \right]}}^{\left[\alpha^{\prime }\right]}$ when α is the knowledge, attitude, and practice layers, respectively; Figure 7d–f represent the correlations between 1 − $q_{i}^{\left[\alpha \right]}$ and 1 − $q_{O_{q}^{\left[\alpha \right]}}^{\left[\alpha^{\prime }\right]}$ when α is the knowledge, attitude, and practice layers, respectively.

As shown in Figure 7, for the positive nodes in the α layer, as the proportion of negative (a–c) or positive (d–f) neighbors increased, the probability of being negative or positive at another layer shows a rising trend. The results indicate a strong positive correlation. The higher the proportion of negative (positive) neighbors, the greater the potential negative (positive) influence on the node. Taking Figure 7b,e for example, for the positive nodes in the attitude layer, as their negative (positive) neighbors increase, the probably of being negative (positive) in other layers increases.

For positive nodes in a certain layer, as negative or positive neighbors increase, the probabilities of being negative or positive, respectively, in the other two layers generally rise, albeit at differing slopes. The difference indicates that neighbors have differing levels of impact on individuals in the other layer. For instance, in Figure 7a, Curve P2 sits above Curve P1, which indicates that, for an individual whose neighbor ratio is constant, the probability of being negative in the practice layer is higher than in the attitude layer. Meanwhile, the slope of Curve P2 (0.783) was greater than that of Curve P1 (0.758) (as shown in Table A3). This implies that, when the number of negative neighbors increase, negative neighbors affect the practice of positive nodes in the knowledge layer more so than their attitude. In sum, the probabilities of being positive or negative at the other layers stems from a node’s own state (intercept) and neighbors’ influence (slope).

If we cross compare the contrasting graphs for the same positive nodes in a certain layer, the differing slopes of corresponding curves, such as Curve P2 and P2′ in Figure 7a,b, suggest that negative and positive neighbors of positive nodes in the knowledge layer have differing levels of impact on behavior. Because the slopes of Curve P1′ and P2′ were greater (as shown in Table A3), this means positive neighbors have a stronger influence than negative neighbors do on positive nodes in the knowledge layer. That is, when the numbers of negative or positive neighbors increase, the positive nodes in the knowledge are more likely to be positively affected.

#### 3.3.3. Neighbors’ Influence on Negative FSWs

To investigate the negative influence of neighbors on negative nodes, we studied FSWs’ performances in other layers. In other words, for a negative FSW in a certain layer, we looked at how her responses (i.e., positive/negative probability in a layer) were correlated with her neighbors’ responses. Let ${\bar{q}}_{i}^{\left[\alpha \right]}$ denote the positive neighbor ratio of node i, as shown in Equation (11).

(11) $${\bar{q}}_{i}^{\left[\alpha \right]}=\frac{{\bar{N}}_{i}^{positive}}{{\bar{N}}_{i}^{positive}+{\bar{N}}_{i}^{negative}}$$

The negative neighbor ratio of negative node  i is 1 − ${\bar{q}}_{i}^{\left[\alpha \right]}$. ${\bar{N}}_{i}^{positive},{\bar{N}}_{i}^{negative}$ denote the number of positive and negative neighbors of node i. Thus, all the negative nodes with the same ${\bar{q}}_{i}^{\left[\alpha \right]}$ value form a subset ${\bar{O}}_{q}^{\left[\alpha \right]}$. Then, let ${\bar{q}}_{{\bar{O}}_{q}^{\left[\alpha \right]}}^{\left[\alpha^{\prime }\right]}$ denote the ratio of being positive in the $\alpha^{\prime }$ layers for the nodes in ${\bar{O}}_{q}^{\left[\alpha \right]}$, as shown in Equation (12). $N_{{\bar{O}}_{q}^{\left[\alpha \right]}}\;$denotes the elements in ${\bar{O}}_{q}^{\left[\alpha \right]}$, and ${\bar{N}}_{{\bar{O}}_{q}^{\left[\alpha \right]}}^{positive},{\bar{N}}_{{\bar{O}}_{q}^{\left[\alpha \right]}}^{negative}$ denote the positive and negative numbers in $\alpha^{’}$ layers for the nodes in${\bar{\;O}}_{q}^{\left[\alpha \right]}$.

(12) $${\bar{q}}_{{\bar{O}}_{q}^{\left[\alpha \right]}}^{\left[\alpha^{\prime }\right]}=\frac{{\bar{N}}_{O_{q}^{\left[\alpha \right]}}^{positive}}{N_{{\bar{O}}_{q}^{\left[\alpha \right]}}}$$

**Figure 8:**
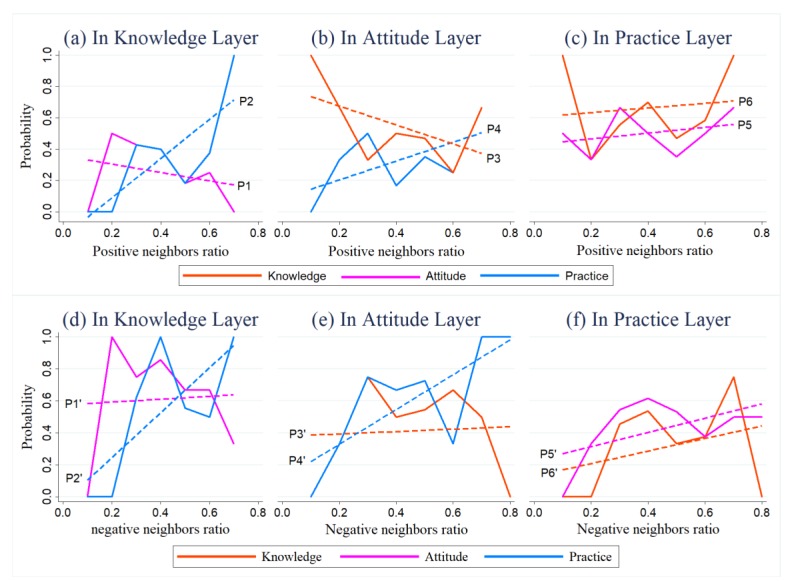
Negative (**a**–**c**) and positive (**d**–**f**) neighbors’ influence on negative FSWs. The solid and dashed lines represent the true values and the linear fitted values the probability of ${\bar{q}}_{{\bar{O}}_{q}^{\left[\alpha \right]}}^{\left[\alpha^{\prime }\right]}$ and $1\;-\;{\bar{q}}_{{\bar{O}}_{q}^{\left[\alpha \right]}}^{\left[\alpha^{\prime }\right]}$, respectively. Please refer to Table A4 for the fitted equations and parameters of the models as well as the results of significant testing. We only provided the conclusions when the fitted lines passed the related significant testing.

Similarly, the negative ratio in the $\alpha^{\prime }$ layers for the nodes in ${\bar{O}}_{q}^{\left[\alpha \right]}$ is 1 − ${\bar{q}}_{{\bar{O}}_{q}^{\left[\alpha \right]}}^{\left[\alpha^{\prime }\right]}$. Figure 8a–c show the correlations between ${\bar{q}}_{i}^{\left[\alpha \right]}$ and ${\bar{q}}_{{\bar{O}}_{q}^{\left[\alpha \right]}}^{\left[\alpha^{\prime }\right]}$, and Figure 8d–f show the correlations between 1 − ${\bar{q}}_{i}^{\left[\alpha \right]}$ and $1\;-\;{\bar{q}}_{{\bar{O}}_{q}^{\left[\alpha \right]}}^{\left[\alpha^{\prime }\right]}$. The *x*-axis represents ${\bar{q}}_{i}^{\left[\alpha \right]}$ (Figure 8a–c) or 1 − ${\bar{q}}_{i}^{\left[\alpha \right]}$ (Figure 8d–f) (i.e., the positive or negative neighbor ratio of negative node i), while the *y*-axis represents ${\bar{q}}_{{\bar{O}}_{q}^{\left[\alpha \right]}}^{\left[\alpha^{\prime }\right]}$ (Figure 8a–c) or $1\;-\;{\bar{q}}_{{\bar{O}}_{q}^{\left[\alpha \right]}}^{\left[\alpha^{\prime }\right]}$ (Figure 8d–f) (i.e., the ratio of being positive or negative in the $\alpha^{\prime }$ layers for the nodes in ${\bar{O}}_{q}^{\left[\alpha \right]}$). Figure 8a–c, respectively, represent the correlations between ${\bar{q}}_{i}^{\left[\alpha \right]}$ and ${\bar{q}}_{{\bar{O}}_{q}^{\left[\alpha \right]}}^{\left[\alpha^{\prime }\right]}$ when α is the knowledge, attitude, and practice layers; Figure 8d–f, respectively, represent the correlations between 1 − ${\bar{q}}_{i}^{\left[\alpha \right]}$ and $1\;-\;{\bar{q}}_{{\bar{O}}_{q}^{\left[\alpha \right]}}^{\left[\alpha^{\prime }\right]}$ when α is the knowledge, attitude, and practice layers.

As we can see from Figure 8, for negative nodes in layer α, as the proportion of positive (negative) neighbor increased, the probability of being positive (negative) in another layer generally shows a rising tendency, which indicates a positive correlation to some degree.

For negative nodes in the attitude or knowledge layers, positive or negative neighbors generally had a more significant influence on their practice as opposed to knowledge and attitude, wherein the negative effects were greater (refer to Table A3 for the slope values), as shown in Figure 8a,b,d,e. Indeed, the linear regression for practice rose sharply, while the others were more stable. For instance, as shown in Figure 8b, as the number of positive neighbors increased, the practice of the negative nodes tended to be more positive, while their attitude remained essentially unchanged. In addition, comparing Figure 8a,d,b,e, the slopes of P2′ and P4′ were greater than those of Curve P2 and P4, respectively. This indicates that the practice of negative nodes in the attitude and knowledge layers was more likely to be affected by negative neighbors.

For negative nodes in the practice layer, when the number of positive neighbors increased, they were less likely to become positive in the attitude and knowledge layers; on the other hand, when the number of negative neighbors increased, they were more likely to be negative in the attitude and knowledge layers. Overall, negative nodes are more likely to be affected by negative neighbors, and the negative influences are greater than the positive ones.

## 4. Discussion

Prior studies and programs among FSWs have reported improvement of HIV knowledge [16,17] and sexual behavioral changes [18,19] by distributing educational materials, providing free condoms, etc. [16]. Those studies largely focused on examining individual attribute characteristics [34,35]. But the mutual influence between peers and acquaintances within a social network has rarely been explored. Based on a multilayer social network architecture, we proposed a probability model to reveal the inter-correlation of FSWs’ responses in KAP as well as the relationships between the individuals and their acquaintances or companions. The constructed model combined the individual’s characteristics and her structural features in the social network to quantify the impacts the peers have on a focal individual using a statistical probability method.

We found that most FSWs have a passive attitude toward HIV/AIDS prevention although they have a certain understanding of HIV/AIDS. In general, there are strong positive correlations at all layers. For positive FSWs, their attitude is the most likely to be negatively affected, followed by knowledge and practice, while for negative FSWs, practice is the most likely to be positively affected, followed by knowledge and attitude. Based on these findings, HIV/AIDS interventions should be tailored to suit FSWs with different KAP scores. For positive FSWs, it is necessary to pay more attention to attitude to help prevent negative influences. For negative FSWs, more attention should be paid to their neighbors and peer education because they are more likely to be influenced by negative neighbors.

In general, negative FSWs exhibit high homophily in knowledge, attitude, and practice with negative neighbors, while positive FSWs exhibit homophily in knowledge with positive neighbors. For positive FSWs in the knowledge layers, positive neighbors are more likely to affect them so they might become positive in all three layers more easily than other nodes. Therefore, programs should make full use of peer education, namely, working with positive FSWs to strengthen HIV/AIDS prevention. As for FSWs with negative Z-scores, they are more likely to be affected by positive neighbors in practice than in knowledge and attitude. So, relevant policies concerning practice to improve the effectiveness of intervention and education programs should be made.

Since the KAP framework describes a kind of sequential or causal relationship, measuring the conditional probability that a FSW positive in knowledge is also positive in attitude or practice provides enough implications. However, a reverse path from practice to knowledge/attitude also gives contributions. In fact, the sequential relationship described by the KAP framework is not an inevitable causal relationship, but a probability relationship. For the FSWs, there are many impact factors that would make their behaviors changed, such as social culture, customs, habits, public opinion, laws and regulations, etc. Therefore, behavioral changes of an FSW does not depend solely on the shift in knowledge or in attitude. In other words, for the positive FSWs in practice, their knowledge and attitude are not necessarily positive. In the future, we will try to quantify the impacts on their behavior caused by knowledge change, as well as other impact factors, to find efficient approaches to make FSWs more positive on behavior.

There is also much future work that may further extend this study. For example, this study collected individual characteristic and network structural information through interviews with 93 FSWs. We assumed the responses were truthful. However, the use of a questionnaire might be subject to social desirability or other systematic biases [44,46,47,48,49,50]. In the future, we could explore more representative and comprehensive datasets with alternative data collection approaches. For example, we can rely on log data or social media data from online communities and platforms of FSWs [51,52]. We could also devote ourselves to interviewing more FSWs at different workplaces and/or in different districts or areas to provide more generalizable conclusions.

## 5. Conclusions

Female sex workers have drawn a lot of attention in the context of HIV/AIDS prevention. In this paper, we used a knowledge, attitude, and practice (KAP) questionnaire to explore the correlations among these three aspects based on the social networks of FSWs. We interviewed FSWs on their KAP in relation to HIV/AIDS and inquired about their social interactions to construct a multi-layer FSW social network. We then analyzed the relationships among the aforementioned factors. We have shown peer influences on a focal FSW in terms of knowledge, attitude, and practice based on the multi-layer FSW social network. We hope that our efforts in this paper can help improve the effectiveness of AIDS/HIV intervention based on social interactions within the FSWs.

## Figures and Tables

**Table 1:** Knowledge, attitude, and practice toward HIV/AIDS transmission and control (*n* = 93). Note that Questions 1 to 3 relate to knowledge; 4 and 5 to attitude; and 6 and 7 to practice.

Questions	Correct or Positive Answer	Correct or Positive, %
1. Can sharing food with an HIV-infected person transmit HIV?	No	59.14%
2. Can shaking hands with an HIV-infected person transmit HIV?	No	63.44%
3. Can having sex without a condom with an HIV-infected person transmit HIV?	Yes	90.32%
4. In the last 12 months, have you received free condoms through free STI treatment?	Yes	1.08%
5. In the last 12 months, have you received free condoms through free HIV/STI counseling and testing?	Yes	1.08%
6. In the last six months, have you been tested for an STI?	Yes	30.11%
7. Even if a client offered more money, I could still insist on using a condom.	Yes	86.02%

**Table 2:** Positive/negative distributions of FSWs.

Categories	K + A + P	K + A	A + P	K + P	K	A	P
Positive FSWs	16	22	5	9	10	6	6
Negative FSWs	19	6	10	6	5	9	22

**Table 3:** Proportion of positive FSWs in different workplaces.

Sub-Layer	Bar or Karaoke Hall	Salon or Beauty Salon	Sauna or Massage Parlor	Nightclubs or Hotels	Other
Knowledge	76%	72.7%	50%	85.7%	44.4%
Attitude	64%	63.6%	0	71.4%	36.1%
Practice	36%	59.1%	50%	71.4%	22.2%

Note that we performed a chi-square test to verify that workplace difference was correlated to the positivity of FSWs. Please see Appendix B (Table A6 and Table A7) for details.

## Appendix A

**Table A1:** Knowledge, attitude, and practice questions on HIV/AIDS transmission, prevention, and control (*n* = 93).

Categories	Questions	Correct or Positive Answer	Correct or Positive, %
Knowledge Layer	1. Can sharing food with an HIV-infected person transmit HIV?	No	59.14%
	2. Can shaking hands with an HIV-infected person transmit HIV?	No	63.44%
	3. Can having sex without a condom with an HIV-infected person transmit HIV?	Yes	90.32%
	4. Can having sex with a condom with an HIV-infected person transmit HIV?	No	53.76%
	5. Can being bitten by a mosquito transmit HIV?	No	32.25%
	6. Can a blood transfusion transmit HIV?	Yes	83.87%
	7. Can sharing needles transmit HIV?	Yes	80.65%
	8. Can an HIV-infected pregnant woman transmit HIV to her fetus?	Yes	79.57%
	9. Have you ever heard of the HIV virus or an illness called AIDS?	Yes	96.77%
	10. Do you personally know anyone who has HIV or has died from AIDS?	Yes	1.08%
	11. Do you think there is a possibility that you yourself will contract HIV?	Yes	12.90%
	12. Do you think a healthy-looking person could be infected with HIV?	Yes	8.60%
	13. Do you think a sexual partner you know well could be infected with HIV/AIDS?	Yes	5.38%
	14. Do you think oral contraceptive pills can prevent HIV transmission?	No	60.21%
	15. Do you think using external contraceptives during sex can prevent HIV transmission?	No	63.44%
	16. Do you think using a male condom during sex can prevent HIV transmission?	Yes	67.74%
	17. Do you think not sharing needles when injecting drugs can prevent HIV transmission?	Yes	62.37%
	18. Do you think using a clean needle when injecting drugs can prevent HIV transmission?	Yes	65.59%
**Attitude Layer**	19. In the last 12 months, have you received free condoms through free STI treatment?	Yes	1.08%
	20. In the last 12 months, have you received free condoms through free HIV/STI counseling and testing?	Yes	1.08%
	21. In the last 12 months, have you received free condoms through free promotional material?	Yes	1.08%
	22. In the last 12 months, have you received free condoms through HIV/AIDS or STI prevention basic skills training?	Yes	1.08%
	23. In the last 12 months, have you received information about HIV/AIDS through a newspaper, magazine, book or pamphlet?	Yes	48.39%
	24. In the last 12 months, have you received information about HIV/AIDS through a health worker, friend or peer?	Yes	50.54%
	25. In the last 12 months, have you received information about HIV/AIDS through television advertisements?	Yes	55.91%
**Practice Layer**	26. In the last six months, have you been tested for an STI?	Yes	30.11%
	27. Even if a client offered more money, I could still insist on using a condom.	Yes	86.02%
	28. Do you have the non-regular sex partner?	No	79.57%
	29. Do you have a regular sex partner?	Yes	56.99%
	30. The last time you had sex with a commercial partner, did you use a condom?	Yes	91.40%
	31. Do you have condoms now?	Yes	82.80%
	32. Are you currently married or living with a sexual partner?	Yes	78.49%

**Table A2:** Fitted equations and parameters for the curves in Figure 4.

Sub-Graph	Lines	Fitted Equations	*R*-Squared
(a)	P1	y=0.093x − 0.484 (0.002***) (0.088**)	0.828
	P2	y=0.072x− 0.412 (0.096**) (0.445)	0.394
(b)	P3	y=0.603x+0.651 (0.222) (0.086**)	0.883
	P4	y=0.149x+0.086 (0.117*) (0.585)	0.966
(c)	P5	y=0.076x +0.102 y=0.047x+0.396	1.000
	P6		1.000
(d)	P1′	y=0.039x+0.216 (0.03***) (0.324)	0.465
	P2′	y=0.039x+0.172 (0.091**) (0.554)	0.316
(e)	P3′	y=0.230x− 0.899 y=0.173x − 0.424	1.00
	P4′		1.00
(f)	P5′	y=0.192x+0.475 (0.708) (0.100*)	0.085
	P6′	y=0.050x+0.283 (0.225) (0.116*)	0.600

Note: The values in brackets are the *p*-values of the *t-*tests in corresponding regression models, in which the *, **, and *** represent the parameters which passed the *t*-test at the 0.05, 0.10 or 0.20 significance levels, respectively. The same for below in Table A3 and Table A4.

**Table A3:** Fitted equations and parameters for the curves in Figure 7.

Sub-Graph	Lines	Fitted Equation	*R*-Squared
(a)	P1	y=0.758x + 0.004 (0.004***) (0.964)	0.772
	P2	y=0.783x + 0.159 (0.835) (0.312)	0.556
(b)	P3	y=0.447x + 0.014 (0.229) (0.938)	0.230
	P4	y=1.180x + 0.036 (0.002***) (0.752)	0.831
(c)	P5	y=0.807x + 0.053 (0.159*) (0.842)	0.301
	P6	y=0.108x + 0.172 (0.828) (0.503)	0.009
(d)	P1′	y=0.929x + 0.196 (0.030***) (0.282)	0.570
	P2′	y=0.983x + 0.006 (0.045***) (0.977)	0.516
(e)	P3′	y=0.406x + 0.621 (0.202) (0.005***)	0.255
	P4′	y=1.115x − 0.054 (0.006***) (0.705)	0.738
(f)	P5′	y=1.157x − 0.024 (0.004***) (0.858)	0.778
	P6′	y=0.953x + 0.185 (0.099)** (0.482)	0.388

**Table A4:** Fitted equations and parameters for the curves in Figure 8.

Sub-Graph	Lines	Fitted Equations	*R*-Squared
(a)	P1	y=−0.267x + 0.385 (0.537) (0.103*)	0.081
	P2	y=1.251x − 0.160 (0.035***) (0.450)	0.622
(b)	P3	y=−0.606x + 0.798 (0.228) (0.010***)	0.274
	P4	y=0.602x + 0.083 (0.157*) (0.628)	0.357
(c)	P5	y=0.186x + 0.429 (0.509) (0.014***)	0.092
	P6	y=0.148x + 0.604 (0. 789) (0.050***)	0.016
(d)	P1′	y=0.089x + 0.575 (0.903) (0.126)	0.003
	P2′	y=1.404x − 0.036 (0.058**) (0.895)	0.544
(e)	P3′	y=0.075x + 0.378 (0.879) (0.162*)	0.004
	P4′	y=1.089x + 0.111 (0.029***) (0.584)	0.575
(f)	P5′	y=0.445x + 0.225 (0.149*) (0.148*)	0.314
	P6′	y=0.394x + 0.129 (0.409) (0.584)	0.116

**Table A5:** Symbols and related implications.

Symbol	Implication
α	Any layer of knowledge, attitude, and practice
$\alpha^{\prime }$	The complementary set of α
$T_{i}^{\left[\alpha \right]}$	The number of positive responds showed by node i at α layer
$u^{\left[\alpha \right]}$	The mean of the number of positive responds showed by node i at α layer
$\sigma^{\left[\alpha \right]}$	The variance of the number of positive responds showed by node i at α layer
$Z_{i}^{\left[\alpha \right]}$	The Z-Score value of node i at α layer
$W_{i}$	The positive weight of node i in the multi-layer FSW social network
$\bar{W_{i}}$	The negative weight of node i in the multi-layer FSW social network
$F_{i}^{\left[\alpha \right]}$	The number of negative responds showed by node i at α layer
$R_{i}^{\left[\alpha \right]}$	The weight of positive node i at α layer
${\bar{R}}_{i}^{\left[\alpha \right]}$	The weight of negative node i at α layer
$n_{i}^{\left[\alpha \right]}$	The impact index from node i negative neighbors at α layer
$s_{i}^{\left[\alpha \right]}$	The impact index received of node i from all of her positive and negative neighbors
$a_{i}^{\left[\alpha \right]}$	$a_{i}^{\left[\alpha \right]}$ = 1 when node i is consisted in positive (negative) group in α layer, otherwise $a_{i}^{\left[\alpha \right]}$= 0
$n^{\left[\alpha \right]}$	The number of questions in α layer
$O^{\left[\alpha \right]}$	The positive (negative) FSWs set in α layer
$O_{g}^{\left[\alpha \right]}$	The subset of FSWs who possess the same number of positive responses in α layer
g	The number of positive (negative) responses of subset $O_{g}^{\left[\alpha \right]}$ in α layer
$\;p\left(a_{i}^{\left[\alpha^{\prime }\right]}/a_{i}^{\left[\alpha \right]}\right)$	The probability of positive (negative) node i remains positive (negative) in $\alpha^{\prime }$ layer
$\;P\left(a_{i}^{\left[\alpha^{\prime }\right]}/a_{i}^{\left[\alpha \right]}\right)$	The probabilities that positive (negative) node i in $O_{g}^{\left[\alpha \right]}$ remains positive (negative) in $\alpha^{\prime }$ layer
$T_{i}$	the overall positive degree of node i
$N_{i}^{positive}$	The number of all positive neighbors of positive node i
$N_{i}^{negative}$	The number of all negative neighbors of positive node i
$q_{i}^{\left[\alpha \right]}$	The negative neighbor ratio of positive node i in α layer
$O_{q}^{\left[\alpha \right]}$	The subset of the positive nodes that possess the same $q_{i}^{\left[\alpha \right]}$ in α layer
$N_{O_{q}^{\left[\alpha \right]}}$	The number of positive nodes in $O_{q}^{\left[\alpha \right]}$
$N_{O_{q}^{\left[\alpha \right]}}^{negative}$	The number of being negative nodes in $O_{q}^{\left[\alpha \right]}$ in $\alpha^{\prime }$ layer
$q_{O_{q}^{\left[\alpha \right]}}^{\left[\alpha^{\prime }\right]}$	The ratio of being negative in $\alpha^{\prime }$ layers for the nodes in $O_{q}^{\left[\alpha \right]}$
${\bar{N}}_{i}^{positive}$	The number of all positive neighbors of negative node i
${\bar{N}}_{i}^{negative}$	The number of all negative neighbors of negative node i
${\bar{q}}_{i}^{\left[\alpha \right]}$	The positive neighbor ratio of negative node i in α layer
${\bar{O}}_{q}^{\left[\alpha \right]}$	The subset of the negative nodes that possess the same ${\bar{q}}_{i}^{\left[\alpha \right]}$ in α layer
$N_{{\bar{O}}_{q}^{\left[\alpha \right]}}$	The number of positive nodes in ${\bar{O}}_{q}^{\left[\alpha \right]}$
${\bar{N}}_{{\bar{O}}_{q}^{\left[\alpha \right]}}^{positive}$	The number of being positive nodes in ${\bar{O}}_{q}^{\left[\alpha \right]}$ in $\alpha^{\prime }$ layer
$q_{{\bar{O}}_{q}^{\left[\alpha \right]}}^{\left[\alpha^{\prime }\right]}$	The ratio of being positive in $\;\alpha^{\prime }$ layers for the nodes in ${\bar{O}}_{q}^{\left[\alpha \right]}$

## Appendix B. Chi-Square Tests for FSWs in Different Workplaces

First, we take the female sex workers (FSWs) in the knowledge layer, for example, to demonstrate the testing process, and then list all the testing results in Table A7. Here, we declare the null hypothesis H_0_ and alternative hypothesis H_1_:
**Hypothesis** **0** **(H_0_):** The workplace difference has no effect on the proportion of positive FSWs in the Knowledge layer.
**Hypothesis** **1** **(H_1_):** The workplace difference has an effect on the proportion of positive FSWs in the Knowledge layer.

Table A6 Gives the contingency table for the chi-square test for the FSWs in the knowledge layer.

**Table A6:** Contingency table for chi-square test.

Workplace	Frequency		Sum
	Positive FSWs	Negative FSWs	
Bar or Karaoke hall	19	6	25
Salon or Beauty salon	16	7	23
Nightclubs or Hotels	6	1	7
Others	16	20	36
Sum	58	35	93

Note: we deleted the workplace of sauna or massage parlor as there were only 2 FSW samples in those workplaces.

Using the contingency table above, we calculated the chi-square values and their statistical significance (*p*-values) for the FSWs in the knowledge layer, as shown in Table A7. Then we obtained the chi-square test results for the FSWs in the attitude and practice layers.

**Table A7:** Results of the chi-square test for the FSWs in the KAP layer.

Sub-Layer	Chi-Square	*p*-Value
Knowledge	9.063	0.028 **
Attitude	6.856	0.076 *
Practice	10.459	0.015 **

Note: *p*-values with * or ** represent that we should reject the null hypothesis H_0_ at the significance level of 0.1 or 0.05. In other words, the type of workplace is correlated to the positivity of FSWs in a certain sub-layer.
